# Osteochondritis dissecans-like lesions of the occipital condyle and cervical articular process joints in a Saddlebred colt horse

**DOI:** 10.1186/s13028-017-0345-5

**Published:** 2017-10-30

**Authors:** Chee Kin Lim, Jan Fletcher Hawkins, Andrea Lynn Vanderpool, Hock Gan Heng, Caroline Cooper Gillespie Harmon, Stephen Dana Lenz

**Affiliations:** 1 Department of Veterinary Clinical Sciences, College of Veterinary Medicine, Purdue University, West Lafayette, IN 47907 USA; 2 Department of Comparative Pathobiology, College of Veterinary Medicine, Purdue University, West Lafayette, IN 47907 USA

**Keywords:** Cervical, Computed tomography, Equine, Osteochondritis dissecans, Radiography

## Abstract

**Background:**

Osteochondritis dissecans (OCD) is a sequela to osteochondrosis, whereby the cartilage superficial to the site of osteochondrosis fractures and gives rise to osteochondral fragments in the affected joint. In this case, both the radiological and computed tomography findings were supportive of classical severe OCD but the histologic findings were not supportive of the diagnosis of OCD.

**Case presentation:**

A 1 year and 6 months old, Saddlebred, colt was presented for evaluation of chronic cervical pain. Standing laterolateral radiographs revealed an osteochondral fragment with corresponding irregular subchondral bone defect at one of the occipital condyle. Computed tomography confirmed the presence of osteochondral fragments at the left occipital condyle and several articular process joints of the cervical spine, with associated subchondral bone defects and sclerosis, suggestive of OCD. However, the lack of ischemic chondronecrosis microscopically was not supportive of a histologic diagnosis of OCD. Therefore, the term ‘OCD-like lesions’ was deemed most appropriate for these cervical lesions.

**Conclusion:**

In the event where imaging features were characteristics of OCD but lack of histologic evidence of ischemic chondronecrosis, the term ‘OCD-like lesion’ is deemed most appropriate.

## Background

The term ‘osteochondritis dissecans’ (OCD) which is widely used in veterinary and human medicine was first introduced by König in 1887 to describe a condition that was associated with the formation of fragments in the joints of human patients [1]. However, this was actually a misnomer and was later changed to osteochondrosis dissecans as inflammation was not a characteristic of the primary lesions [2]. In horses, the term osteochondritis dissecans is still maintained when osteochondritis dissecans and synovitis are diagnosed concurrently [3]. Due to the ubiquity of the usage of the term OCD in equine literature, the authors decided to maintain this terminology for this case.

## Case presentation

A 350 kg, 1 year and 6 months old, Saddlebred, colt was admitted for evaluation of cervical pain of approximately 1 year duration. Physical examination by the referring veterinarian had revealed severe signs of cervical pain without obvious neurological deficits. According to the referring veterinarian, there were no obvious radiographic lesions observed on standing cervical radiographs. At admission, physical examination was within normal limits with the exception of resistance to turning of the neck. A neurological examination was performed and there were no obvious neurological deficits. Based on the historical findings of cervical pain standing, repeated cervical radiography was recommended to the owner.

## Diagnostic imaging

**Fig. 1:**
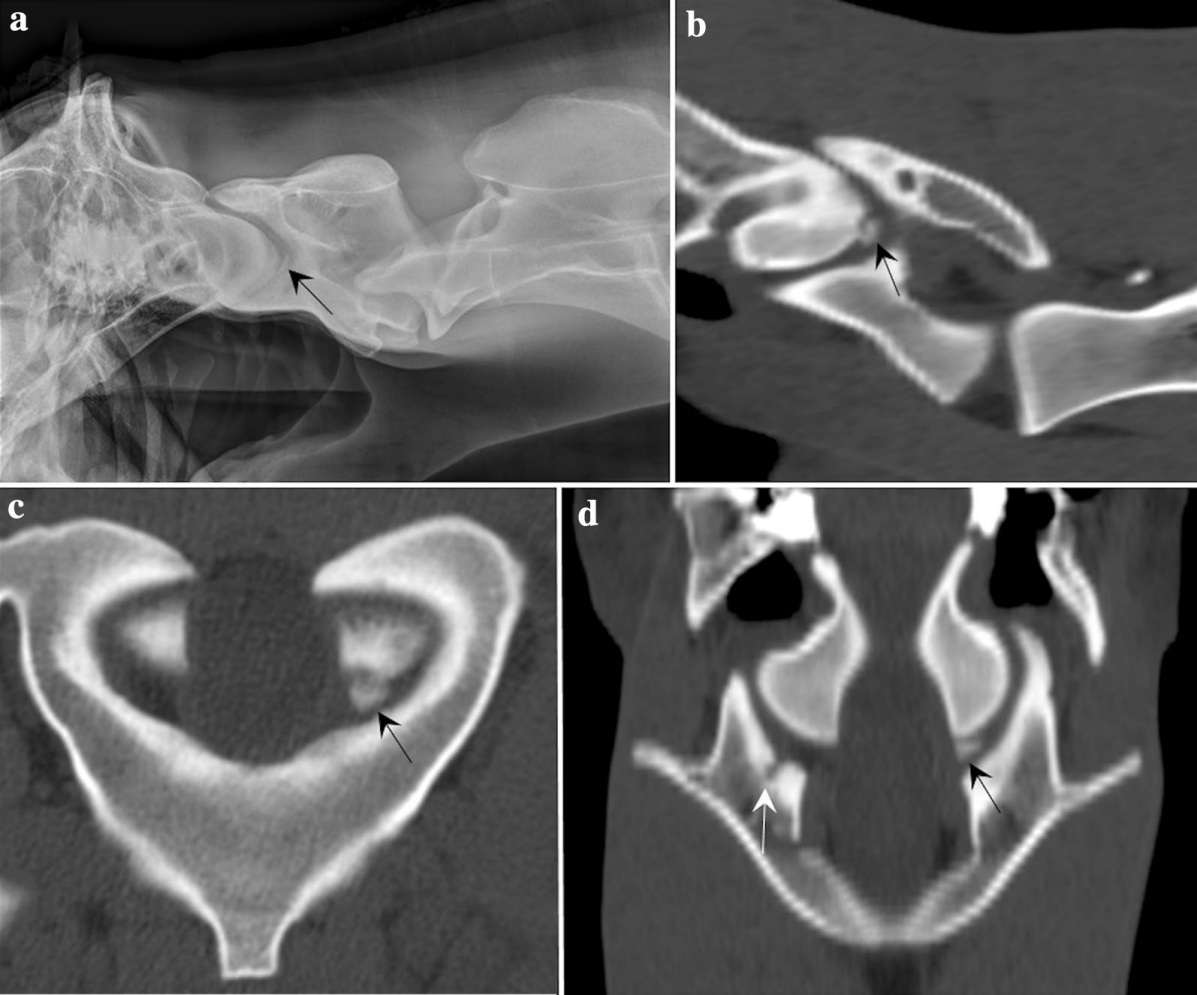
Osteochondritis dissecans-like lesion at the occipital condyle. **a** Laterolateral radiographs and **b** sagittal, **c** transverse and **d** dorsal multiplanar reconstructed CT images of the cranial cervical spine showing a small, discrete osteochondral fragment (black arrow) at the caudoventral margin of the left occipital condyle and a concave defect at the right cranial articular process of the atlas with associated subchondral bone sclerosis (white arrow)

**Fig. 2:**
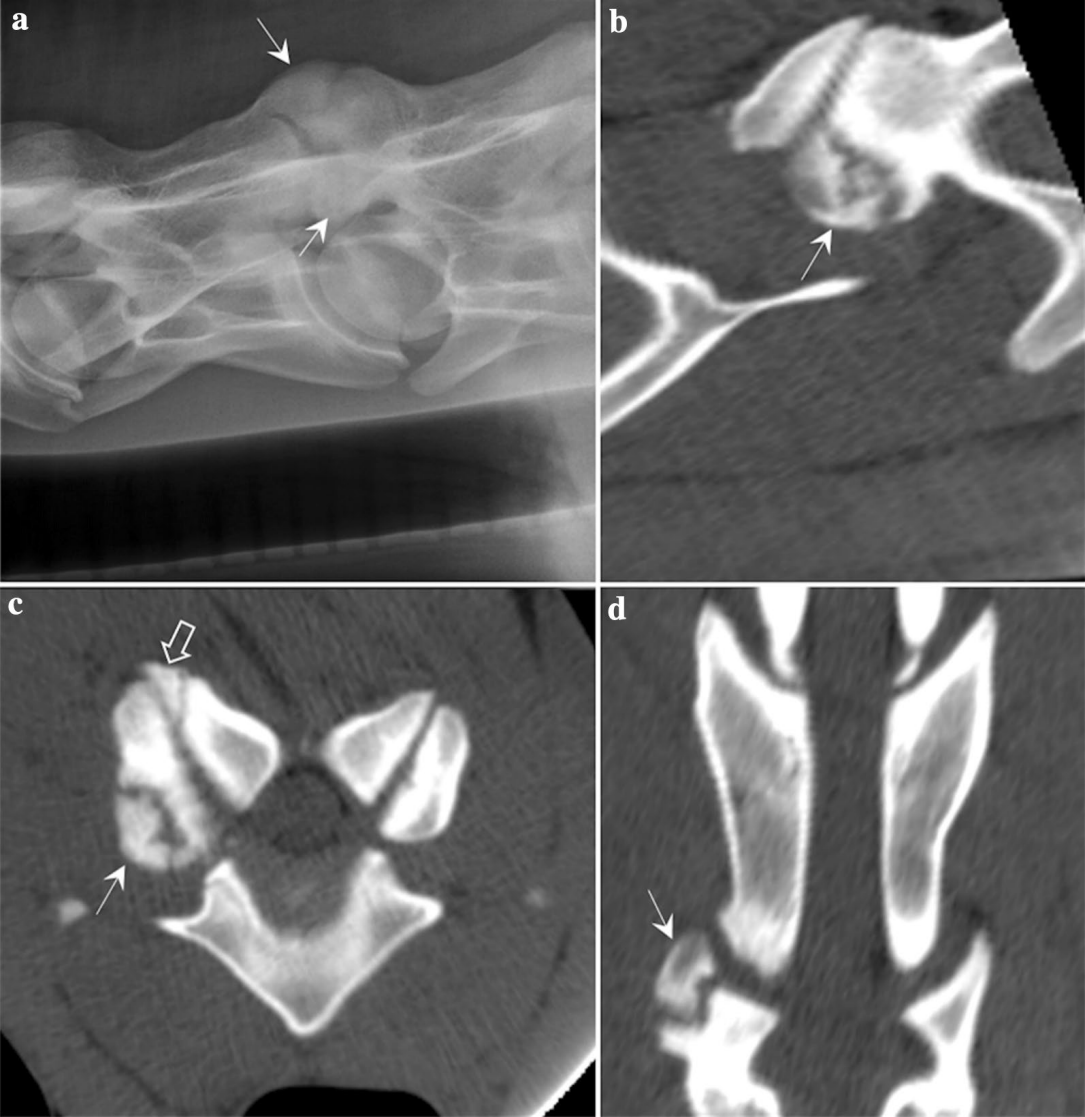
Osteochondritis dissecans-like lesion at C5–C6 articular process joint. **a** Laterolateral radiographs showing bulbous osteophyte (white arrows) was present at the C5–C6 articular process joint (APJ) with obliteration of the joint space. **b** Sagittal, **c** transverse and **d** dorsal multiplanar reconstructed CT images showing a large and irregularly marginated osteochondral fragment with few smaller fragments at the right cranial articular process of C6 (white arrows) and a smaller osteochondral fragment is seen at the lateral edge of the right caudal articular process of C5 (open white arrow), with associated subchondral bone defect and sclerosis

Standing laterolateral radiography of the entire cervical spine was performed (Control-X Medical. Inc, Milestone HF Generator, Columbus, Ohio) with the settings of 80 kVp and 10–20 mAs. A poorly defined elongated osseous fragment (7.5 mm × 2 mm) was present between the occipital condyles and the atlas, with a corresponding mild irregular subchondral bone defect on one of the occipital condyles (Fig. 1a). A bulbous osteophyte was present at the C5–C6 articular process joint (APJ) with obliteration of the joint space and mild cranioventral encroachment of the articular process (Fig. 2a).

The radiological findings were discussed with the owner and based on the poor prognosis for soundness, the owner elected to donate the horse. To further evaluate the radiological findings, a computed tomographic (CT) scan of the cervical spine was performed. The horse was anesthetized in a routine fashion and placed under inhalational anesthesia. The horse was positioned in right lateral recumbency with the head extended on a custom designed table for equine computed tomography.

**Fig. 3:**
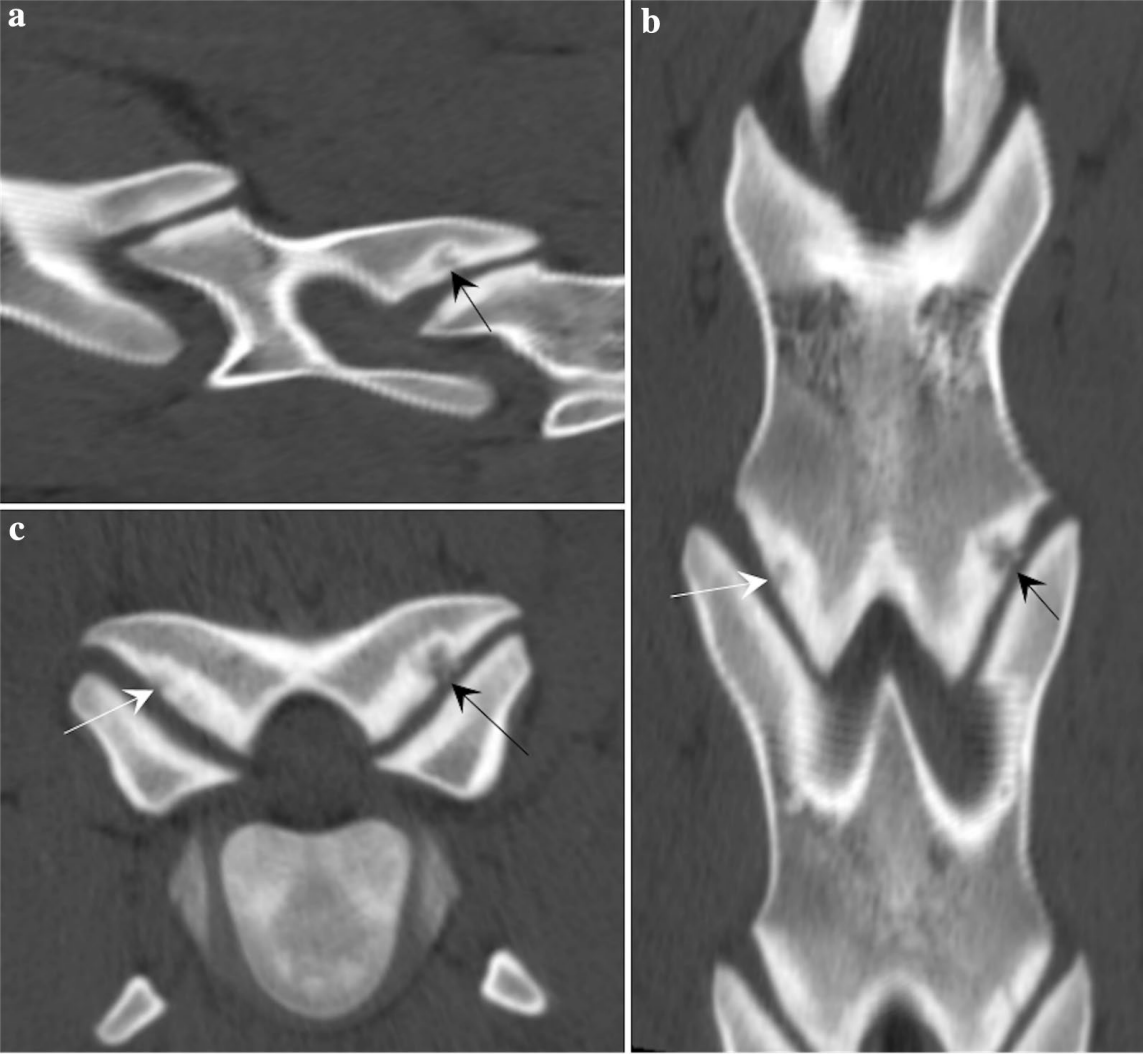
Osteochrondritis dissecans-like lesion at caudal C3 articular processes. **a** Sagittal, **b** transverse and **c** dorsal multiplanar reconstructed CT images of C3 and C4 cervical vertebrae, showing an irregular concave defect at the left C3 caudal articular process with associated subchondral sclerosis and a very small osteochondral fragment (black arrow). Similar but smaller subchondral defect and sclerosis was noted at the right C3 caudal articular process (white arrow)

Helical CT scan of the cervical spine from caudal skull to C5–C6 intervertebral disc space was performed (GE LightSpeed VCT 64-Slice, Milwaukee, Wisconsin) with the following parameters: 140 kV, 98 mAs, 2.5 mm slices with reconstruction interval of 1.25 mm, tube rotation time 1 s and pitch of 1.0. A small osteochondral fragment was present at the ventral margin of the left occipital condyle, with associated subchondral sclerosis (Fig. 1b–d). A small and focal concave defect at the right cranial articular process of the atlas with associated subchondral sclerosis was also noted (Fig. 1d). Few irregular concave defects were present at the left C3 caudal articular process with associated subchondral sclerosis and a very small osteochondral fragment (Fig. 3a–c). Two smaller concave defects were noted at the right C3 caudal articular process. A small and focal concave defect was present at the left C4 caudal articular process. A large irregularly marginated osteochondral fragment with few smaller osteochondral fragments were seen at the right cranial articular process of C6 and a smaller osteochondral fragment was seen at the lateral edge of the right caudal articular process of C5, with associated subchondral sclerosis (Fig. 2b–d). Mild irregularity was present at the left caudal articular process of C5 and cranial articular process of C6 (Fig. 2c). Based on the radiological and computed tomographic findings, the imaging diagnosis of osteochondritis dissecans of the left occipital condyle and multiple sites of cervical spine APJs was made. There was no evidence of extradural cord compression.

## Outcome, gross necropsy and histopathologic findings

At the completion of the CT scan, the horse was euthanized with an overdose of barbiturates and submitted for necropsy.

**Fig. 4:**
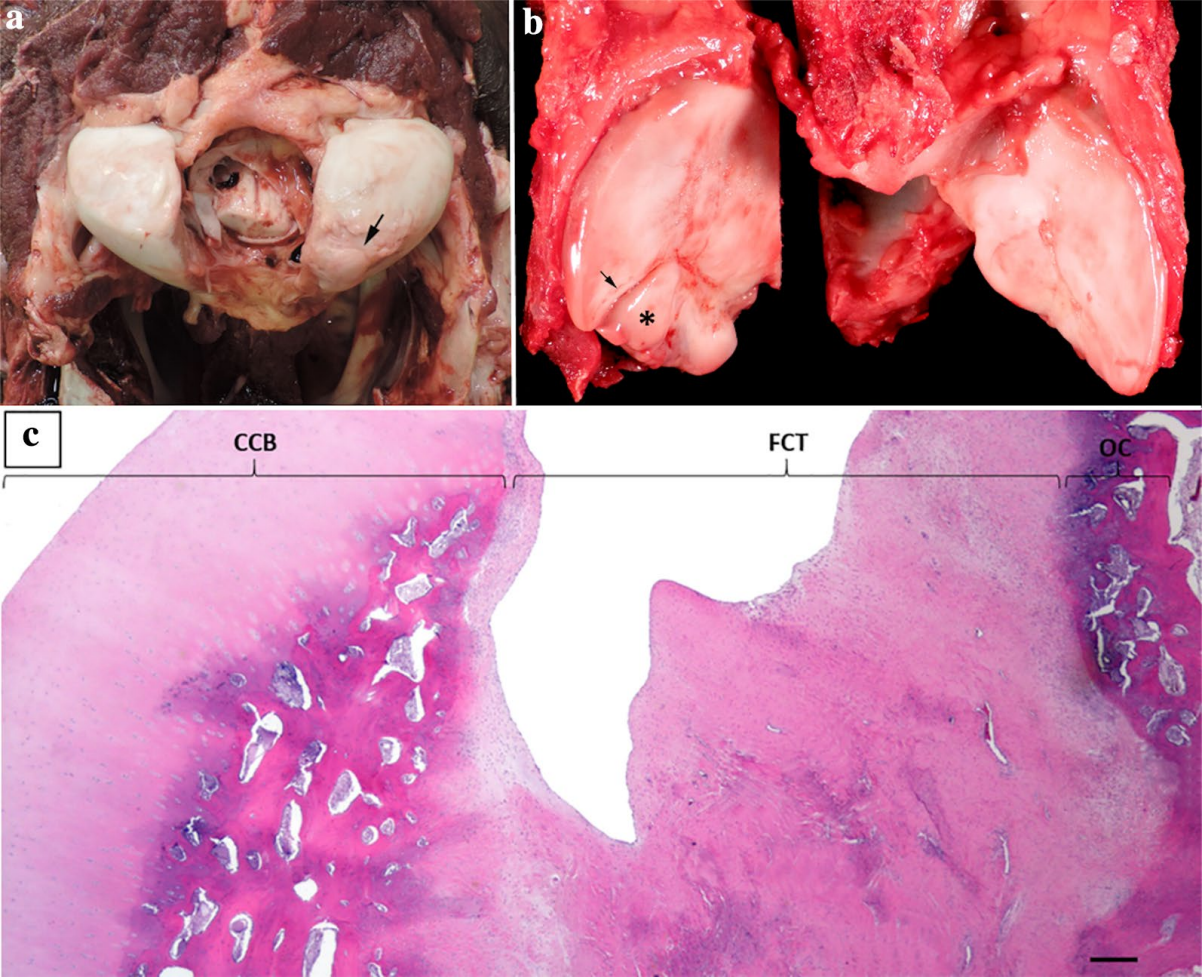
Necropsy and histopathological findings of the occipital condyle and right C6 articular process. **a** A loosely attached cartilage fragment is adhered to the articular cartilage of the left occipital condyle (large black arrow). **b** A cartilage-covered fragment (*) expands the ventrolateral surface of the right cranial articular facet of C6 and bordered by a short linear fissure (small black arrow). **c** 20× magnification; H&E stain of cartilage-capped bone from the occipital condyle. Fibrous connective tissue (FCT) connects a fragment of cartilage-capped bone (CCB) to the occipital condyle (OC). Black bar = 200 µm

Gross necropsy findings included a 1 cm × 1 cm loosely attached cartilage-covered fragment adhered to the ventromedial articular cartilage of the left occipital condyle (Fig. 4a). Cervical vertebral APJ lesions were located at the caudal articular process of C3, caudal left articular process of C4, caudal articular process of C5, and cranial and caudal articular processes of C6. Affected cervical vertebral articular processes were traversed by multiple linear, interlacing cartilage-covered fissures. The right C6 cranial articular process was focally expanded by a 1.5 cm cartilage-covered fragment on its ventrolateral surface (Fig. 4b). Flexion of the cervical vertebral resulted in compression of the spinal cord at C3–C4, C4–C5, and C5–C6 intervertebral disc spaces.

Histopathologically, the cartilage surface of affected articular processes was uneven and the subchondral bone was sclerotic. Shallow fissures extended to subchondral bone and were lined by attenuated cartilage containing clusters of proliferative chondrocytes. In the left occipital condyle, the cartilage-covered bone fragment maintained a fibrous connective tissue connection to the occipital condyle. Subchondral bone in the fragment and within the occipital condyle below the fragment was sclerotic (Fig. 1c). In the right cranial C6 articular process, a deep fissure extending into subchondral bone was filled with fibrocartilage and separated articular cartilage from a 1.5 cm irregular bony fragment. This fragment was covered by a very thin layer of fibrocartilage on its articular surface that lacked normal chondrocytes (Fig. 1d). Lack of ischemic chondronecrosis precluded diagnosis of active OCD in the occipital condyle and the right cranial C6 articular process, as well as other affected APJs. Transverse and longitudinal sections of the spinal cord corresponding to the previously described sites of gross cord compression showed no evidence of neurodegeneration. The pathologic diagnosis based on the gross and histopathologic findings was compatible with cervical vertebral instability and diffuse spondyloarthritis. Microscopic lesions represented various phases of bone and cartilage repair following local injury. The attached bony fragment on the occipital condyle was most likely representative of a developmental anomaly, or less likely a traumatic lesion.

The consensual diagnosis between the radiologists and pathologists for this horse was osteochondritis dissecans-like lesions involving the occipital condyle and multiple cervical APJs.

## Discussion and conclusions

This was an unusual and interesting case as the imaging findings were highly suggestive of OCD of the occipital condyle and multiple cervical APJs and yet, the lack of evidence of ischemic chondronecrosis microscopically was not supportive of a histologic diagnosis of OCD. Hence, these lesions were termed ‘OCD-like lesions’. Similar ‘OCD-like lesion’ have only been reported once in a 11-month old dog, involving the intercondylar fossa of the left femur [4]. Ostechondrosis refers to a focal disturbance in endochondral ossification [5–7]. Osteochondritis dissecans is a sequela to osteochondrosis, whereby the cartilage superficial to the site of osteochondrosis fractures and gives rise to osteochondral fragments in the affected joint [7]. Although osteochondrosis has been considered to be a result of primary disease of collagen from 1997 to 2007, but from 2008 onwards, there is a paradigm shift towards considering vascular failure as the primary cause to osteochondrosis [7]. In pigs and horses, disturbance in ossification has been confirmed to be caused by failure of blood supply to epiphyseal growth cartilage, leading to ischemic chondronecrosis [7–10]. The factors that determine whether a horse will manifest clinical signs associated with osteochondrosis remains obscure [8]. The predilected sites of OCD in horses includes the femoropatellar, tarsocrural, fetlock and scapulohumeral joints [7, 11]. The equine cervical APJs are also subject to developmental defects, osteochondrosis, fractures and degenerative joint disease [12–14]. Osteochondrosis have also been reported to involve the cervical vertebrae [15–20]. When skeletal lesions of the articular process joints were examined in horses with confirmed cervical vertebral stenotic myelopathy, osteochondrosis was identified as one of the most common lesion histologically [16]. Osteochondrosis of the occipital condyles has been reported and diagnosed at necropsy in one case report but was not detected on cervical radiographs [21].

Imaging diagnosis of OCD in horses in clinical cases are frequently made based on radiography and occasionally CT, with or without necropsy or histopathology [7, 11, 15–20, 22, 23]. Depending on the joint involved, classical radiological changes of osteochondrosis or OCD include presence of discrete osteochondral fragments, flattening or concave defect in the articular surface and subchondral lucency or sclerosis with or without secondary osteoarthritis [11]. However, in order to detect an osseous lesion radiographically, at least 30–50% variation in bone density must present [24, 25]. Also, summation of bony densities and superimposition of bony contours can sometimes make detection of subchondral bone lesions difficult [25]. The use of CT in equine sports medicine has become increasingly popular as CT is able to detect osteoarthritis, subchondral bone lesions and osseous cysts-like lesions in horses without bony superimposition [26]. Both conventional CT and micro-CT have proven to be able to detect experimentally induced osteochondrosis lesions in horses [22, 23]. In this case, both the radiological and CT findings were supportive of classical severe OCD given the presence of discrete osteochondral fragments, subchondral bone defects and sclerosis of the left occipital condyle and APJs even though the histologic findings suggested otherwise.

Histologically, osteochondrosis have three classifications: osteochondrosis latens, osteochondrosis manifesta and OCD [7]. Osteochondrosis latens is a focal area of necrosis of the epiphyseal cartilage, and osteochondrosis manifesta is when the necrosis extends into the subchondral bone. Osteochondritis dissecans is the most severe manifestation with loss of integrity between the cartilage and bone, resulting in formation of loose cartilaginous flap or loose osteochondral body and characterized by ischemic chondronecrosis due to failure of blood supply to growth cartilage [2, 7]. Since there was no evidence of ischemic chondronecrosis detected in any of the affected cervical sites in this case, the histologic diagnosis of OCD cannot be established. Given that the imaging findings showed classical OCD lesions while histologic findings suggested otherwise, the authors therefore suggested the consensual term of ‘OCD-like lesions’ in this case.

The exact cause of the ‘OCD-like lesions’ observed in this case is unknown. Trauma may be an underlying cause even though one may expect history of trauma. Alternatively, this may also be an unusual form of mild but chronic osteochondrosis in a growing young horse. One study have shown that excessive biomechanical load on certain articular surfaces of immature horses can result in formation of early osteochondrosis lesions [27]. However, in these immature horses, the repair process often follows almost immediately after formation of osteochondrosis lesions and may result in complete regression of many initial lesions [22]. As the horse had been exhibiting cervical pain for over a year, there is possibility that the ‘OCD–like lesions’ in this case have already underwent reparative phase whereby the necrotic growth cartilage has sustained creeping substitution and replaced by viable bone tissue and hence the lack of evidence of ischemic chondronecrosis.

The treatment of OCD lesions in the horse can be conservative or surgical [28]. Surgery is chosen for the majority of horses diagnosed with intra-articular osteochondral fragments [8, 28]. However, the surgical treatment of OCD lesions in horses with cervical spinal abnormalities is limited. The only surgical treatment available for horses diagnosed with cervical instability related to OCD is interbody fusion techniques [29, 30]. For the case reported here, the interesting finding was that the horse had no evidence of neurologic deficits grossly but had a primary clinical complaint of neck pain. However, the extensive nature of the osteochondral defects confirmed with diagnostic imaging, gross and histologic findings made the prognosis for long-term soundness poor, necessitating euthanasia.

In conclusion, ‘OCD-like lesion’ can occur in young horses and should be considered as a differential diagnosis for horses presented with cervical pain. The authors suggest that in the event where imaging features were characteristics of OCD but lack of histologic evidence of ischemic chondronecrosis, the term ‘OCD-like lesion’ is deemed most appropriate.

## Abbreviations

APJ: articular process joint
CT: computed tomography
OCD: osteochondritis dissecans
